# 4-Chloro-*N*-(3-methyl­phen­yl)benzene­sulfonamide

**DOI:** 10.1107/S1600536811019416

**Published:** 2011-05-28

**Authors:** K. Shakuntala, Sabine Foro, B. Thimme Gowda

**Affiliations:** a Department of Chemistry, Mangalore University, Mangalagangotri 574 199, Mangalore, India; b Institute of Materials Science, Darmstadt University of Technology, Petersenstrasse 23, D-64287 Darmstadt, Germany

## Abstract

In the crystal of the title compound, C_13_H_12_ClNO_2_S, the N—H bond is *anti* to the *meta*-methyl group in the aniline ring. The C—SO_2_—NH—C torsion angle is −57.6 (2)°. The sulfonyl and aniline benzene rings are tilted relative to each other by 84.7 (1)°. The crystal structure features inversion-related dimers linked by pairs of N—H⋯O hydrogen bonds.

## Related literature

For hydrogen-bonding modes of sulfonamides, see; Adsmond & Grant (2001 ▶). For our study of the effect of substituents on the structures of *N*-(ar­yl)-amides, see: Gowda *et al.* (2004 ▶), on the structures of *N*-(ar­yl)aryl­sulfonamides, see: Gowda *et al.* (2010 ▶); Nirmala *et al.* (2009 ▶); Shakuntala *et al.* (2011 ▶) and on the structures of *N*-(ar­yl)methane­sulfonamides, see: Gowda *et al.* (2007 ▶).
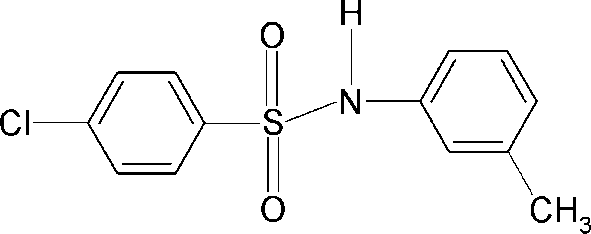


## Experimental

### 

#### Crystal data

C_13_H_12_ClNO_2_S
*M* *_r_* = 281.75
Monoclinic, 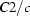

*a* = 14.202 (1) Å
*b* = 14.561 (1) Å
*c* = 13.271 (1) Å
β = 97.292 (9)°
*V* = 2722.2 (3) Å^3^
*Z* = 8
Mo *K*α radiation
μ = 0.43 mm^−1^
*T* = 293 K
0.48 × 0.44 × 0.40 mm

#### Data collection

Oxford Diffraction Xcalibur diffractometer with a Sapphire CCD detector
Absorption correction: multi-scan (*CrysAlis RED*; Oxford Diffraction, 2009 ▶) *T*_min_ = 0.821, *T*_max_ = 0.848
5706 measured reflections
2777 independent reflections
2186 reflections with *I* > 2σ(*I*)
*R*_int_ = 0.012

#### Refinement

*R*[*F* ^2^ > 2σ(*F* ^2^)] = 0.043
*wR*(*F* ^2^) = 0.119
*S* = 1.05
2777 reflections
167 parameters
1 restraint
H atoms treated by a mixture of independent and constrained refinement
Δρ_max_ = 0.44 e Å^−3^
Δρ_min_ = −0.45 e Å^−3^

### 

Data collection: *CrysAlis CCD* (Oxford Diffraction, 2009 ▶); cell refinement: *CrysAlis RED* (Oxford Diffraction, 2009 ▶); data reduction: *CrysAlis RED*; program(s) used to solve structure: *SHELXS97* (Sheldrick, 2008 ▶); program(s) used to refine structure: *SHELXL97* (Sheldrick, 2008 ▶); molecular graphics: *PLATON* (Spek, 2009 ▶); software used to prepare material for publication: *SHELXL97*.

## Supplementary Material

Crystal structure: contains datablocks I, global. DOI: 10.1107/S1600536811019416/ds2114sup1.cif

Structure factors: contains datablocks I. DOI: 10.1107/S1600536811019416/ds2114Isup2.hkl

Supplementary material file. DOI: 10.1107/S1600536811019416/ds2114Isup3.cml

Additional supplementary materials:  crystallographic information; 3D view; checkCIF report

## Figures and Tables

**Table 1:** Hydrogen-bond geometry (Å, °)

*D*—H⋯*A*	*D*—H	H⋯*A*	*D*⋯*A*	*D*—H⋯*A*
N1—H1*N*⋯O2^i^	0.84 (2)	2.11 (2)	2.942 (2)	172 (2)

Symmetry code: (i) 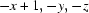.

**Fig. 1.**
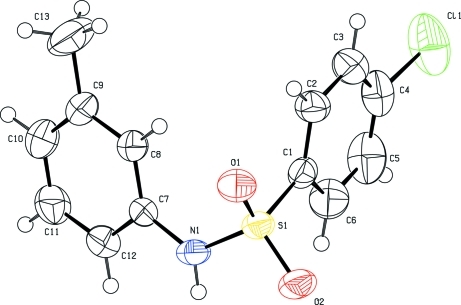
Molecular structure of (I), showing the atom labelling scheme and displacement ellipsoids are drawn at the 50% probability level.

**Fig. 2.**
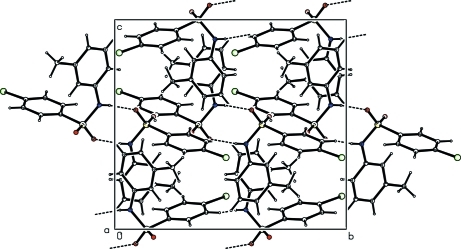
Molecular packing of (I) with hydrogen bonding shown as dashed lines.

## supplementary crystallographic information

### Comment

The sulfonamide moieties are the constituents of many biologically important compounds. The hydrogen bonding preferences of sulfonamides has been investigated (Adsmond & Grant, 2001). As a part of studying the substituent effects on the structures of this class of compounds (Gowda *et al.*, 2004, 2007, 2010; Nirmala *et al.*, 2009, Shakuntala *et al.*, 2011), in the present work, the crystal structure of 4-chloro-*N*-(3-methylphenyl)-benzenesulfonamide (I) has been determined (Fig.1). In the title compound, the N—C bond in the C—SO_2_—NH—C segment has *gauche* torsions with respect to the S═O bonds. Furthermore, the N—H bond is *anti* to the *meta*-methyl group in the anilino ring, similar to that observed in 4-methyl-*N*-(3-methylphenyl)-benzenesulfonamide (II) (Nirmala *et al.*, 2009), but in contrast to the *syn* conformation observed with respect to the *meta*-methyl groups in *N*-(3-methylphenyl)-benzenesulfonamide (III) (Gowda *et al.*, 2010)

The molecule is twisted at the S atom with the C—SO_2_—NH—C torsion angle of -57.6 (2)°, compared to the values of 56.7 (3)° in (II), 55.8 (2)° (molecule 1) and -58.4 (3)° (molecule 2) in the two molecules of (III), and -53.8 (3)° and -63.4 (3)° in the two independent molecules of 4-chloro-*N*-(phenyl)-benzenesulfonamide (IV)(Shakuntala *et al.*, 2011).

The sulfonyl and the anilino benzene rings are tilted relative to each other by 84.7 (1)° in (I), compared to the values of 83.9 (1)° in (II), 67.9 (1)° (molecule 1) and 68.6 (1)° (molecule 2) in (III), and 69.1 (1)° and 82.6 (1)° in the two independent molecules of (IV).

The packing of molecules in the crystal *via* intermolecular N—H···O hydrogen bonds (Table 1) is shown in Fig. 2.

### Experimental

The solution of chlorobenzene (10 ml) in chloroform (40 ml) was treated dropwise with chlorosulfonic acid (25 ml) at 0 ° C. After the initial evolution of hydrogen chloride subsided, the reaction mixture was brought to room temperature and poured into crushed ice in a beaker. The chloroform layer was separated, washed with cold water and allowed to evaporate slowly. The residual 4-chlorobenzenesulfonylchloride was treated with *m*-toluidine in the stoichiometric ratio and boiled for ten minutes. The reaction mixture was then cooled to room temperature and added to ice cold water (100 ml). The resultant 4-chloro-*N*-(3-methylphenyl)-benzenesulfonamide was filtered under suction and washed thoroughly with cold water. It was then recrystallized to constant melting point from dilute ethanol. The compound was characterized by recording its infrared and NMR spectra.

Needle like colorless single crystals used in X-ray diffraction studies were grown in ethanolic solution by slow evaporation at room temperature.

### Refinement

The H atom of the NH group was located in a difference map and later restrained to the distance N—H = 0.86 (2) Å. The other H atoms were positioned with idealized geometry using a riding model with the aromatic C—H = 0.93Å and the methyl C—H = 0.96 Å. All H atoms were refined with isotropic displacement parameters (set to 1.2 times of the *U*_eq_ of the parent atom).

### Crystal data

**Table:**

C_13_H_12_ClNO_2_S	*F*(000) = 1168
*M**_r_* = 281.75	*D*_x_ = 1.375 Mg m^−^^3^
Monoclinic, *C*2/*c*	Mo *K*α radiation, λ = 0.71073 Å
Hall symbol: -C 2yc	Cell parameters from 2392 reflections
*a* = 14.202 (1) Å	θ = 2.7–27.8°
*b* = 14.561 (1) Å	µ = 0.43 mm^−^^1^
*c* = 13.271 (1) Å	*T* = 293 K
β = 97.292 (9)°	Prism, colourless
*V* = 2722.2 (3) Å^3^	0.48 × 0.44 × 0.40 mm
*Z* = 8	

### Data collection

**Table:**

Oxford Diffraction Xcalibur diffractometer with a Sapphire CCD detector	2777 independent reflections
Radiation source: fine-focus sealed tube	2186 reflections with *I* > 2σ(*I*)
graphite	*R*_int_ = 0.012
Rotation method data acquisition using ω scans	θ_max_ = 26.4°, θ_min_ = 2.7°
Absorption correction: multi-scan (*CrysAlis RED*; Oxford Diffraction, 2009)	*h* = −17→16
*T*_min_ = 0.821, *T*_max_ = 0.848	*k* = −18→18
5706 measured reflections	*l* = −16→16

### Refinement

**Table:**

Refinement on *F*^2^	Primary atom site location: structure-invariant direct methods
Least-squares matrix: full	Secondary atom site location: difference Fourier map
*R*[*F*^2^ > 2σ(*F*^2^)] = 0.043	Hydrogen site location: inferred from neighbouring sites
*wR*(*F*^2^) = 0.119	H atoms treated by a mixture of independent and constrained refinement
*S* = 1.05	*w* = 1/[σ^2^(*F*_o_^2^) + (0.0632*P*)^2^ + 1.8143*P*] where *P* = (*F*_o_^2^ + 2*F*_c_^2^)/3
2777 reflections	(Δ/σ)_max_ = 0.008
167 parameters	Δρ_max_ = 0.44 e Å^−^^3^
1 restraint	Δρ_min_ = −0.45 e Å^−^^3^

### Special details

**Table:**

Experimental. CrysAlis RED (Oxford Diffraction, 2009) Empirical absorption correction using spherical harmonics, implemented in SCALE3 ABSPACK scaling algorithm.
Geometry. All e.s.d.'s (except the e.s.d. in the dihedral angle between two l.s. planes) are estimated using the full covariance matrix. The cell e.s.d.'s are taken into account individually in the estimation of e.s.d.'s in distances, angles and torsion angles; correlations between e.s.d.'s in cell parameters are only used when they are defined by crystal symmetry. An approximate (isotropic) treatment of cell e.s.d.'s is used for estimating e.s.d.'s involving l.s. planes.
Refinement. Refinement of *F*^2^ against ALL reflections. The weighted *R*-factor *wR* and goodness of fit *S* are based on *F*^2^, conventional *R*-factors *R* are based on *F*, with *F* set to zero for negative *F*^2^. The threshold expression of *F*^2^ > σ(*F*^2^) is used only for calculating *R*-factors(gt) *etc*. and is not relevant to the choice of reflections for refinement. *R*-factors based on *F*^2^ are statistically about twice as large as those based on *F*, and *R*- factors based on ALL data will be even larger.

### Fractional atomic coordinates and isotropic or equivalent isotropic displacement parameters (Å^2^)

**Table:**

	*x*	*y*	*z*	*U*_iso_*/*U*_eq_	
C1	0.53559 (14)	0.23409 (14)	0.05005 (15)	0.0406 (5)	
C2	0.57647 (15)	0.31911 (14)	0.04598 (18)	0.0491 (5)	
H2	0.6336	0.3256	0.0194	0.059*	
C3	0.53266 (18)	0.39513 (17)	0.0813 (2)	0.0593 (6)	
H3	0.5600	0.4530	0.0788	0.071*	
C4	0.44869 (19)	0.3845 (2)	0.11999 (18)	0.0605 (7)	
C5	0.40852 (18)	0.3008 (2)	0.1274 (2)	0.0730 (8)	
H5	0.3523	0.2950	0.1560	0.088*	
C6	0.45174 (18)	0.2240 (2)	0.0922 (2)	0.0644 (7)	
H6	0.4247	0.1662	0.0967	0.077*	
C7	0.67268 (14)	0.08513 (13)	0.18508 (15)	0.0388 (4)	
C8	0.73805 (14)	0.15662 (14)	0.19410 (16)	0.0442 (5)	
H8	0.7430	0.1949	0.1389	0.053*	
C9	0.79595 (16)	0.17110 (16)	0.28509 (18)	0.0522 (5)	
C10	0.78847 (19)	0.11271 (18)	0.36573 (19)	0.0617 (6)	
H10	0.8272	0.1215	0.4269	0.074*	
C11	0.72408 (19)	0.04155 (17)	0.35625 (19)	0.0611 (6)	
H11	0.7202	0.0024	0.4110	0.073*	
C12	0.66545 (16)	0.02758 (15)	0.26704 (18)	0.0500 (5)	
H12	0.6214	−0.0200	0.2616	0.060*	
C13	0.8658 (2)	0.2492 (2)	0.2948 (2)	0.0907 (10)	
H13A	0.8476	0.2935	0.3423	0.109*	
H13B	0.8664	0.2779	0.2297	0.109*	
H13C	0.9281	0.2262	0.3184	0.109*	
N1	0.61358 (14)	0.06654 (12)	0.09302 (14)	0.0510 (5)	
H1N	0.5746 (16)	0.0231 (14)	0.092 (2)	0.061*	
O1	0.67240 (11)	0.16746 (10)	−0.03772 (11)	0.0512 (4)	
O2	0.51716 (12)	0.09095 (11)	−0.06921 (12)	0.0610 (5)	
Cl1	0.38998 (7)	0.48058 (7)	0.15793 (7)	0.1027 (4)	
S1	0.58782 (4)	0.13761 (3)	−0.00050 (4)	0.04315 (18)	

### Atomic displacement parameters (Å^2^)

**Table:**

	*U*^11^	*U*^22^	*U*^33^	*U*^12^	*U*^13^	*U*^23^
C1	0.0383 (10)	0.0444 (11)	0.0372 (10)	−0.0031 (8)	−0.0019 (8)	0.0028 (9)
C2	0.0448 (11)	0.0439 (12)	0.0593 (13)	−0.0027 (9)	0.0093 (10)	−0.0030 (10)
C3	0.0589 (14)	0.0483 (13)	0.0702 (16)	0.0051 (11)	0.0061 (12)	−0.0041 (12)
C4	0.0637 (15)	0.0735 (17)	0.0444 (12)	0.0243 (13)	0.0070 (11)	0.0019 (12)
C5	0.0540 (15)	0.103 (2)	0.0668 (16)	0.0160 (15)	0.0266 (13)	0.0172 (16)
C6	0.0540 (14)	0.0701 (17)	0.0708 (16)	−0.0093 (13)	0.0150 (12)	0.0166 (14)
C7	0.0395 (10)	0.0315 (9)	0.0440 (11)	0.0032 (8)	−0.0004 (8)	−0.0019 (8)
C8	0.0463 (11)	0.0412 (11)	0.0433 (11)	−0.0026 (9)	−0.0017 (9)	0.0033 (9)
C9	0.0493 (12)	0.0527 (12)	0.0508 (12)	−0.0051 (10)	−0.0086 (10)	−0.0008 (11)
C10	0.0654 (15)	0.0674 (16)	0.0470 (13)	0.0000 (12)	−0.0129 (11)	0.0024 (12)
C11	0.0754 (16)	0.0580 (14)	0.0482 (13)	0.0013 (13)	0.0012 (12)	0.0140 (12)
C12	0.0521 (12)	0.0405 (11)	0.0570 (13)	−0.0014 (9)	0.0049 (10)	0.0048 (10)
C13	0.089 (2)	0.100 (2)	0.0737 (19)	−0.0454 (18)	−0.0238 (16)	0.0067 (18)
N1	0.0607 (12)	0.0336 (9)	0.0535 (11)	−0.0148 (8)	−0.0127 (9)	0.0027 (8)
O1	0.0560 (9)	0.0485 (8)	0.0505 (9)	−0.0052 (7)	0.0128 (7)	−0.0060 (7)
O2	0.0759 (11)	0.0490 (9)	0.0514 (9)	−0.0178 (8)	−0.0183 (8)	−0.0017 (7)
Cl1	0.1129 (7)	0.1136 (7)	0.0848 (6)	0.0634 (6)	0.0249 (5)	−0.0053 (5)
S1	0.0500 (3)	0.0366 (3)	0.0402 (3)	−0.0082 (2)	−0.0044 (2)	−0.0017 (2)

### Geometric parameters (Å, °)

**Table:**

C1—C2	1.371 (3)	C8—H8	0.9300
C1—C6	1.386 (3)	C9—C10	1.381 (3)
C1—S1	1.761 (2)	C9—C13	1.505 (3)
C2—C3	1.381 (3)	C10—C11	1.377 (4)
C2—H2	0.9300	C10—H10	0.9300
C3—C4	1.366 (4)	C11—C12	1.373 (3)
C3—H3	0.9300	C11—H11	0.9300
C4—C5	1.354 (4)	C12—H12	0.9300
C4—Cl1	1.736 (3)	C13—H13A	0.9600
C5—C6	1.386 (4)	C13—H13B	0.9600
C5—H5	0.9300	C13—H13C	0.9600
C6—H6	0.9300	N1—S1	1.6216 (19)
C7—C12	1.387 (3)	N1—H1N	0.839 (16)
C7—C8	1.390 (3)	O1—S1	1.4236 (15)
C7—N1	1.418 (3)	O2—S1	1.4380 (15)
C8—C9	1.388 (3)		
			
C2—C1—C6	120.1 (2)	C8—C9—C13	120.0 (2)
C2—C1—S1	120.29 (16)	C11—C10—C9	120.5 (2)
C6—C1—S1	119.59 (18)	C11—C10—H10	119.7
C1—C2—C3	119.9 (2)	C9—C10—H10	119.7
C1—C2—H2	120.0	C12—C11—C10	120.9 (2)
C3—C2—H2	120.0	C12—C11—H11	119.6
C4—C3—C2	119.3 (2)	C10—C11—H11	119.6
C4—C3—H3	120.4	C11—C12—C7	119.3 (2)
C2—C3—H3	120.4	C11—C12—H12	120.4
C5—C4—C3	121.7 (2)	C7—C12—H12	120.4
C5—C4—Cl1	118.8 (2)	C9—C13—H13A	109.5
C3—C4—Cl1	119.5 (2)	C9—C13—H13B	109.5
C4—C5—C6	119.5 (2)	H13A—C13—H13B	109.5
C4—C5—H5	120.2	C9—C13—H13C	109.5
C6—C5—H5	120.2	H13A—C13—H13C	109.5
C5—C6—C1	119.4 (2)	H13B—C13—H13C	109.5
C5—C6—H6	120.3	C7—N1—S1	126.09 (14)
C1—C6—H6	120.3	C7—N1—H1N	118.7 (18)
C12—C7—C8	120.01 (19)	S1—N1—H1N	112.3 (18)
C12—C7—N1	117.75 (18)	O1—S1—O2	118.42 (10)
C8—C7—N1	122.22 (18)	O1—S1—N1	110.01 (10)
C9—C8—C7	120.3 (2)	O2—S1—N1	104.79 (9)
C9—C8—H8	119.9	O1—S1—C1	107.66 (9)
C7—C8—H8	119.9	O2—S1—C1	108.95 (10)
C10—C9—C8	119.0 (2)	N1—S1—C1	106.42 (10)
C10—C9—C13	120.9 (2)		
			
C6—C1—C2—C3	−1.8 (4)	C9—C10—C11—C12	−0.6 (4)
S1—C1—C2—C3	176.87 (18)	C10—C11—C12—C7	1.2 (4)
C1—C2—C3—C4	0.0 (4)	C8—C7—C12—C11	−0.7 (3)
C2—C3—C4—C5	2.0 (4)	N1—C7—C12—C11	177.6 (2)
C2—C3—C4—Cl1	−176.41 (19)	C12—C7—N1—S1	161.80 (17)
C3—C4—C5—C6	−2.0 (4)	C8—C7—N1—S1	−19.9 (3)
Cl1—C4—C5—C6	176.4 (2)	C7—N1—S1—O1	58.7 (2)
C4—C5—C6—C1	0.1 (4)	C7—N1—S1—O2	−172.95 (19)
C2—C1—C6—C5	1.8 (4)	C7—N1—S1—C1	−57.6 (2)
S1—C1—C6—C5	−176.92 (19)	C2—C1—S1—O1	1.6 (2)
C12—C7—C8—C9	−0.4 (3)	C6—C1—S1—O1	−179.70 (18)
N1—C7—C8—C9	−178.6 (2)	C2—C1—S1—O2	−127.99 (18)
C7—C8—C9—C10	0.9 (3)	C6—C1—S1—O2	50.7 (2)
C7—C8—C9—C13	−179.3 (2)	C2—C1—S1—N1	119.52 (19)
C8—C9—C10—C11	−0.4 (4)	C6—C1—S1—N1	−61.8 (2)
C13—C9—C10—C11	179.8 (3)		

### Hydrogen-bond geometry (Å, °)

**Table:**

*D*—H···*A*	*D*—H	H···*A*	*D*···*A*	*D*—H···*A*
N1—H1N···O2^i^	0.84 (2)	2.11 (2)	2.942 (2)	172 (2)

Symmetry codes: (i) −*x*+1, −*y*, −*z*.

## References

[bb1] Adsmond, D. A. & Grant, D. J. W. (2001). *J. Pharm. Sci.* **90**, 2058–2077. 10.1002/jps.1157 11745765

[bb2] Gowda, B. T., Foro, S. & Fuess, H. (2007). *Acta Cryst.* E**63**, o2597.

[bb3] Gowda, B. T., Foro, S., Nirmala, P. G. & Fuess, H. (2010). *Acta Cryst.* E**66**, o434. 10.1107/S1600536810002291 PMC2979874 21579849

[bb4] Gowda, B. T., Svoboda, I. & Fuess, H. (2004). *Z. Naturforsch. Teil A*, **55**, 845–852.

[bb5] Nirmala, P. G., Gowda, B. T., Foro, S. & Fuess, H. (2009). *Acta Cryst.* E**65**, o3208. 10.1107/S1600536809049332 PMC2972154 21578916

[bb6] Oxford Diffraction (2009). *CrysAlis CCD* and *CrysAlis RED* Oxford Diffraction Ltd, Yarnton, England.

[bb7] Shakuntala, K., Foro, S. & Gowda, B. T. (2011). *Acta Cryst.* E**67**, o1252. 10.1107/S1600536811015108 PMC3089100 21754543

[bb8] Sheldrick, G. M. (2008). *Acta Cryst.* A**64**, 112–122. 10.1107/S0108767307043930 18156677

[bb9] Spek, A. L. (2009). *Acta Cryst.* D**65**, 148–155. 10.1107/S090744490804362X PMC2631630 19171970

